# Dry season prevalence of *Plasmodium falciparum* in asymptomatic gambian children, with a comparative evaluation of diagnostic methods

**DOI:** 10.1186/s12936-022-04184-9

**Published:** 2022-06-07

**Authors:** Jason P. Mooney, Sophia M. DonVito, Maimuna Jahateh, Haddy Bittaye, Christian Bottomley, Umberto D’Alessandro, Eleanor M. Riley

**Affiliations:** 1 Institute of Immunology and Infection Research, School of Biological Sciences, University of Edinburgh, Ashworth Laboratories, Kings Buildings, Charlotte Auerbach Rd, Edinburgh, EH9 3FL UK; 2 Medical Research Council Unit in The Gambia at the London School of Hygiene and Tropical Medicine, Fajara, The Gambia; 3 Department of Infectious Disease Epidemiology, London School of Hygiene and Tropical Medicine, London, UK

**Keywords:** Malaria, *Plasmodium*, Subclinical, Asymptomatic, Diagnosis, Gambia

## Abstract

**Background:**

Subclinical infection with *Plasmodium falciparum* remains highly prevalent, yet diagnosing these often low-density infections remains a challenge. Infections can be subpatent, falling below the limit of detection for conventional thick-film microscopy and rapid diagnostic testing (RDT). In this study, the prevalence of subclinical *P. falciparum* infections in school-aged children was characterised at the start of the dry season in the Upper River Region of The Gambia in 2017/2018, with a goal to also compare the utility of different diagnostic tools.

**Methods:**

In a cross-sectional survey of children living in 29 villages on the south bank of the Gambia river (median age of 10 years), matched microscopy, rapid diagnostic test (RDT, detecting histidine-rich protein 2) and polymerase chain reaction (PCR, targeting either 18S rRNA or *var* gene acidic terminal sequence) were used to determine the prevalence of patent and subpatent infections and to compare the performance of the different diagnostic methods.

**Results:**

The prevalence of *var* gene acidic terminal sequence (*var*ATS) qPCR-detectable infections was 10.2% (141/1381) with a median density of 3.12 parasites/µL. Malaria prevalence was highly heterogeneous across the region, ranging from < 1% to ~ 40% prevalence in different village clusters. Compared to *var*ATS, 18S rRNA PCR detected fewer low-density infections, with an assay sensitivity of 50% and specificity of 98.8%. Parasite prevalence in the cohort was 2.9% by microscopy and 1.5% by RDT. Compared to *var*ATS qPCR, microscopy and RDT had sensitivities of 11.5% and 9.2%, respectively, although both methods were highly specific (> 98%). Samples that were positive by all three tests (*var*ATS qPCR, RDT and microscopy) had significantly higher parasite densities (median = 1705 parasites/µL) than samples that were positive by *var*ATS qPCR only (median = 2.4 parasites/µL).

**Conclusions:**

The majority of subclinical malaria infections in school-aged children were of extremely low parasite density and detectable only by ultra-sensitive PCR analysis. Understanding the duration of these low density infections, their physiological impact and their contribution to sustained parasite transmission is necessary to inform malaria elimination strategies.

**Supplementary information:**

The online version contains supplementary material available at 10.1186/s12936-022-04184-9.

## Background

There were an estimated 241 million clinical cases of malaria, globally, in 2020 [1] but the overall burden of malaria is undoubtedly much higher than these estimates suggest given the large proportion of subclinical (asymptomatic) infections among people with acquired clinical immunity living in endemic areas [2]. Subclinical malaria infections go largely untreated as infected individuals rarely come to the attention of health care providers and their infections would, in many cases, only be detectable highly sensitive PCR [2, 3], rather than cheaper and more widely available rapid diagnostic tests (RDTs) or microscopy. Moreover, *Plasmodium falciparum*-infected red blood cells can persist for many months in the spleen [4], bone marrow [5] and other deep tissues [6] serving as a potential reservoir for infection of mosquitoes and onward transmission, especially in areas of seasonal transmission [7, 8].

The prevalence and relative burden of subclinical infections varies widely by region, climate and age [9–11]. In The Gambia, once an area of intense seasonal malaria transmission, longstanding malaria control interventions have reduced the overall prevalence of infection [12] leading to an increasingly heterogeneous distribution of malaria with pockets of residual transmission [13]. Over 50% of infections are now subclinical and more than 30% are subpatent (i.e. detectable by PCR, but not by microscopy) [13]. Furthermore, subpatent infections are more prevalent among school-aged children and adults than younger children, and subpatently infected children are more likely to be anaemic than uninfected children [14]. School-aged children may thus represent an important reservoir of infection and be at risk of potential complications of persistent infection, including anaemia and invasive bacterial disease [15].

In this study, the prevalence of asymptomatic *P. falciparum* infection in school-aged children at the start of the dry season in the Upper River Region of The Gambia in 2017/18 was characterised, with a goal to also compare the sensitivity and specificity of commonly used diagnostic techniques in this population.

## Methods

### Study design and sample collection

A cross-sectional survey of *P. falciparum* infection, among children aged 8–15 years residing in villages in the Upper River Region (URR) of The Gambia, was undertaken between the 11th of December 2017 and the 18th of January 2018 (38 days) [16]. Briefly, 1650 children in good general health, and with a body temperature < 38 °C (as determined using a non-contact, handheld infrared thermometer on the forehead; Hylogy MD-H6), were recruited at village community centres. Additional exclusion criteria included: participation in an ongoing interventional study (within 1 month or during study period); any history, or evidence at screening, of systemic conditions such as cardiovascular, pulmonary, renal, hepatic, neurological, dermatological, endocrine, malignant, infectious, immunodeficiency, psychiatric and other disorders, which could compromise health of the participant during the study or interfere with the interpretation of the study results (including HIV infection, sickle cell disease, functional asplenia, G6PD deficiency or α-thalassaemia); use of immunosuppressive or immune modifying drugs, or antibiotics at study onset or expected use of such during the study period.

In addition to body temperature, height, weight, sex, age, and village of residence were recorded. Pulse (beats per minute, BPM) and oxygen saturation (%) were measured with a pulse oximeter (MeasurPro OX250) and finger prick blood samples were obtained for malaria microscopy (Giemsa-stained thick blood films), rapid diagnosis by lateral flow assay for *P. falciparum* histidine-rich protein 2 (*Pf*HRP2; SD BIOLINE Malaria Ag *P.f*, Abbott) and, for *P. falciparum* PCR and qPCR analysis, dried blood spots were collected onto filter paper (Whatman 3MM CHR, Scientific Laboratory Supplies #CHR1040, UK) and stored in air tight plastic bags with desiccant (0.5 g sachet, GeeJay Chemicals, UK).

### ***Plasmodium falciparum*** diagnostic PCR

For each individual, 3 × 3 mm diameter dried blood spots were punched into a single well of a 96 deep-well plate, digested in 20 µL of proteinase K and 180 µL of ATL buffer solution and extracted using QIAamp 96 DNA QIAcube HT Kits (Qiagen). Extracted DNA was stored at − 70 °C and defrosted at 4 °C prior to use. *Plasmodium falciparum* diagnostic PCR was then performed in two stages, using the same DNA elution for both assays. 18S rRNA nested PCR was conducted (in the MRC laboratories in the Gambia) on the first 788 samples collected. Subsequently, all eluted DNA samples were shipped to the UK and analysed by *var*ATS qPCR.

### 18S rRNA nested PCR

A subgroup of samples were tested for 18S ribosomal RNA as described previously [17, 18] and using a validated protocol established at the molecular diagnostic unit (SOP-QUA-001v3.0) of The Medical Research Council Unit The Gambia at the London School of Hygiene & Tropical Medicine (MRCG@LSHTM). This PCR approach first amplifies *Plasmodium spp*. (nest 1) followed by a second round of PCR (nest 2) targeting *P. falciparum*. DNA samples were analysed in 96-well plates with controls including DNA from *P. falciparum* strain 3D7 as a positive control and uninfected blood and DNA-free wells as negative controls. Samples were run in total reaction volume of 15 µL [1.5 µL reaction buffer (Thermopol), 0.12 µL Taq DNA Polymerase (5 U/µL), 7.88 µL ultrapure water, 0.3 µL dNTPs, 4 µL extracted DNA template, 0.6 µL of each primer at 0.4 µM (rPLU6 = 5′-TTAAAATTGTTGCAGTTAAAACG-3′, and rPLU5new = 5′-CYTGTTGTTGCCTTAAACTTC-3′). Thermocycler conditions were denaturation at 94 °C for 3 min, 24 cycles of 30 s denaturation at 94 °C and annealing and elongation at 58 °C for 30 s and 72 °C for 45 s, and then held at 72 °C for 5 min. This resulted in a PCR product size of approximately 1200 bp. Next, a second ‘nested’ PCR reaction was performed in which samples were run in 15 µL total reaction volume [1.5 µL reaction buffer (Thermopol), 0.12 µL Taq DNA Polymerase (5 U/µL), 11.18 µL ultrapure water, 1 µL PCR product from the previous reaction (‘nest 1’), 0.45 µL of each primer at 0.3 µM (rFAL1–5′-TTAAACTGGTTTGGGAAAACCAAATATATT-3′, and rFAL2–5′-ACACAATAGACTCAATCATGACTACCCGTC-3′). Thermocycler conditions were denaturation at 94 °C for 3 min, 29 cycles of 30 s denaturation at 94˚C and annealing and elongation at 60 °C for 30 s and 72 °C for 45 s, and a final step at 72 °C for 5 min. This resulted in a 205 bp PCR product. PCR products were visualized by electrophoresis (QIAxcel, Qiagen) according to the manufacturer’s instructions and classified as either positive (infected) or negative (uninfected).

### *var*ATS qPCR

For definitive diagnosis, qPCR for the *var* gene acidic terminal sequence (*var*ATS) of *P. falciparum* was performed as described previously [19]. Thawed DNA samples and 10-fold dilutions of a universal standard (NIBSC code 04/176; final concentrations of 1 × 10^8^ to 1 × 10^1^ IU/mL, equivalent to 4.7 × 10^4^ to 4.7 × 10^−3^ parasites/µL) were run in duplicate on a Roche LightCycler 480 II in 384-well plates in total reaction volumes of 12 µL [9 µL mastermix (0.5 µL PCR-grade water, 1.0 µL (0.8µM) *var*ATS forward primer (5′-CCCATACACAACCAAYTGGA), 1.0 µL (0.8µM) *var*ATS reverse primer (5′-TTCGCACATATCTCTATGTCTAT), 0.5 µL (0.4µM) *var*ATS probe (5′-[6FAM]TRTTCCATAAATGGT[BHQ1] and 6.0 µL (1×) Agilent Brilliant III Ultra-Fast qPCR Master Mix, Cat. #600880)] and 3 µL of sample or standard. Thermocycler conditions were pre-incubation at 50 °C for 2 min, denaturation at 95˚C for 10 min, 45 cycles of 15-s denaturation at 95 °C, 1 min annealing and elongation at 55 °C with data acquisition, and a final cooling step at 40 °C for 30 s.

Samples were deemed positive for *P. falciparum* if both replicates were detectable at C_t_ < 40 cycles. Discrepant samples (where only 1 of 2 replicates was detected at C_t_ < 40) were run again in duplicate; only samples that were positive in both replicates on the same plate were deemed infected. In total, eleven 384-well plates were assayed, each with the NIBSC/WHO standard run at 10-fold dilutions in duplicate.

### Data management and statistical analysis

Field data were collected and stored using REDcap data management software [20]. Electronic data were exported to MS Excel for analysis. Malaria prevalence was calculated for each diagnostic test (PCR, RDT and microscopy) and compared to the gold standard of *var*ATS qPCR using McNemar’s test. Test sensitivity was also estimated within strata of *var*ATS qPCR parasite load (< 1, 1–10, 11–100, 101–1000 and > 1000 parasites/µL). All statistical analyses were performed using GraphPad Prism (v.9.1.0). A *p* value of < 0.05 was considered statistically significant.

## Results

**Fig. 1:**
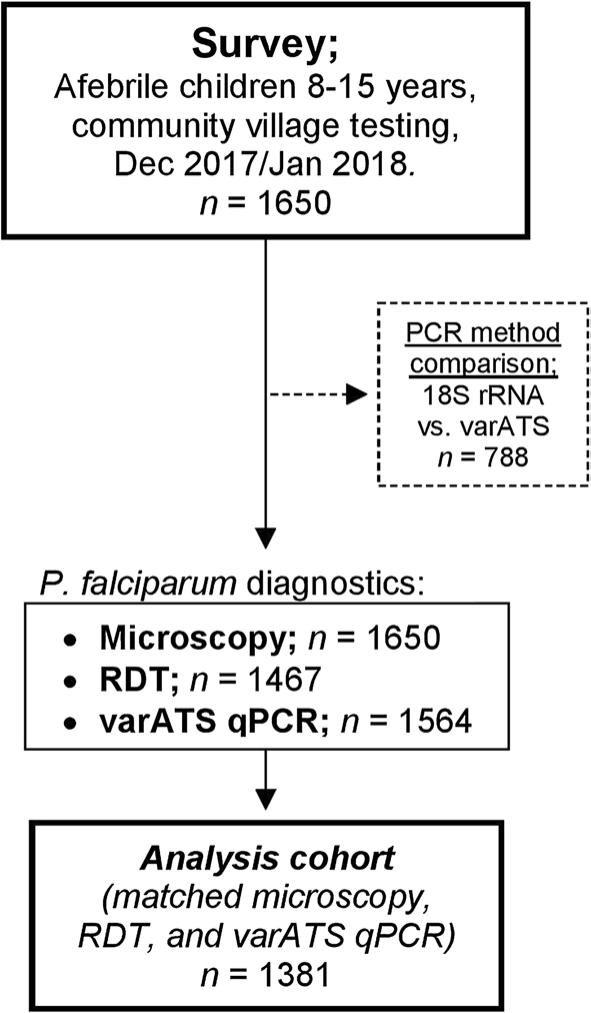
Study design. A cross-sectional survey was conducted at the beginning of the dry season, from December 2017 to January 2018, in the Upper River Region of The Gambia. 1650 children were recruited and blood sampled by finger prick. *Plasmodium falciparum* was detected by microscopy of thick blood films (*n* = 1650), rapid diagnostic test (RDT, *n* = 1467), or quantitative PCR for the *var* gene acidic terminal sequence (*var*ATS) (*n* = 1564). The cohort for analysis consists of children for which results from all three tests were available (*n* = 1381). The performance of *var*ATS qPCR was compared with that of PCR for *P. falciparum* 18S ribosomal RNA in a subgroup of children (*n* = 788) (Fig. 3)

Of the 1650 children initially enrolled, matched microscopy, RDT and *var*ATS qPCR data were available for 1381 children; this group formed the primary analysis cohort for the comparison of diagnostic methods and determination of prevalence of patent and subpatent infections in asymptomatic children (Fig. 1). Whilst microscopy data were available for all children, some RDT data were lost due to an error in the electronic data capture method (*n* = 183) and some blood spot DNA samples were lost in transit to the UK (*n* = 86). Importantly, the analysis cohort did not differ significantly from the complete cohort in terms of demographic or anthropometric parameters (Table 1), indicating that the missing data were unlikely to have introduced significant bias into the analyses. The median age of the analysis cohort was 10 years (IQR 9–12), 51% were male (*n* = 704) and all were afebrile as a condition of enrolment (median temperature = 36.9 °C; IQR 36.7–37.1).

**Fig. 2:**
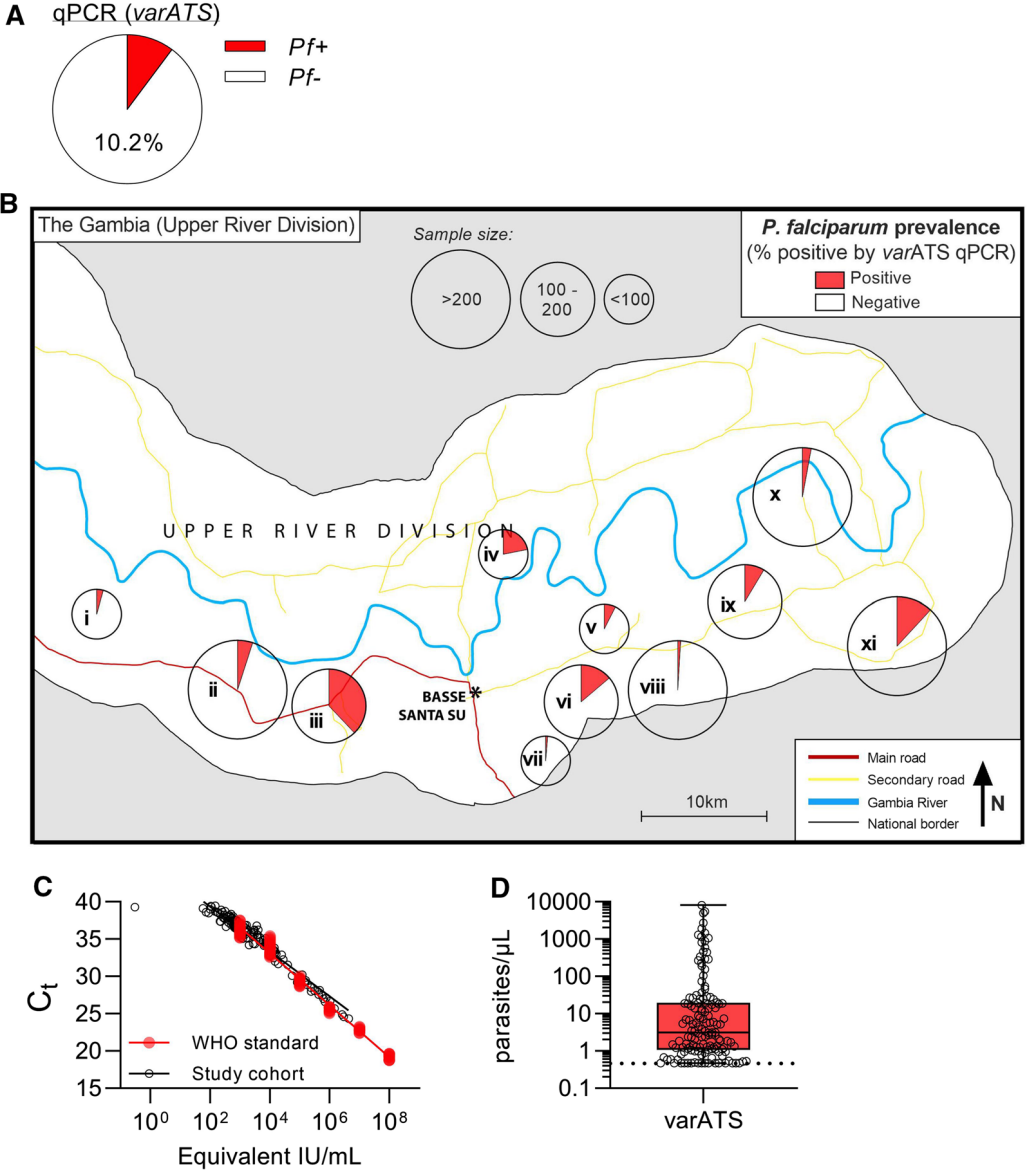
Prevalence and density of *P. falciparum* infection in asymptomatic Gambian children.** A** Prevalence of malaria in the cohort as determined by *var*ATS qPCR. **B** Prevalence is also reported by local village clusters (see Table 2; Additional file 1: Fig. S1 for detailed information). **C** *var*ATS PCR cycle threshold (Ct) values for study cohort and 10-fold dilutions of the NIBSC/WHO International Standard (NIBSC code 04/176), plotted against parasite density (international units, IU, per mL). **D** Parasite density (parasites/µL) was determined by qPCR using the NIBSC/WHO standard, wherein 1 × 10^8^ IU/mL is equal to 4.7 × 10^4^ parasites/µL [19]. Cohort median parasite burden = 3.12 parasites/µL, IQR 1.076–19.110. Dotted line represents LLOQ, 0.47 parasites/µL

Taking the analysis cohort as a whole, the prevalence of *var*ATS qPCR-detectable infections was 10.2% (*n* = 141) (Fig. 2A). There was a tendency for *var*ATS positivity rates to decline over the period of recruitment: children positive by *var*ATS tended to be recruited significantly earlier in the study than *var*ATS negative children (median 16 vs. 28 days, respectively, *p* = 0.0005) (Table 1). This would be consistent with rapid resolution of infections once mosquito populations (and thus transmission) decline in the dry season, but geographical confounding cannot be ruled out as prevalence varied markedly by village/village cluster and villages were sampled sequentially from west to east as the study progressed (Fig. 2B; Additional file 1: Fig. S1), ranging from < 1% to ~ 40% (Table 2).

Next, C_t_ values were plotted against the NIBSC/WHO standard to determine parasite densities (Fig. 2C). On all PCR plates, the standard dilution equivalent to 0.47 parasites/µL (average C_t_ value of 36.2) was the lowest concentration that was positive in both replicates and was deemed the lower limit of quantification (LLOQ). Sixteen patients whose samples were reliably positive but for which parasite density could not be determined (both replicates detected with C_t_ values below 40.0 but greater than 36.2) were assigned the LLOQ value for subsequent quantitative analyses. Overall, parasite density was low in this asymptomatic cohort (median = 3.12 parasites/µL; IQR 1.076–19.110) (Fig. 2D).

There was no significant difference between *var*ATS positive and negative children in terms of age, weight, height, body temperature, pulse rate or oxygen saturation but a significantly higher proportion of male participants (84/704, 11.9%) than female participants (57/677, 8.4%; χ^2^ = 4.64, *p* = 0.031) were parasite positive by *var*ATS (Table 1).

**Fig. 3:**
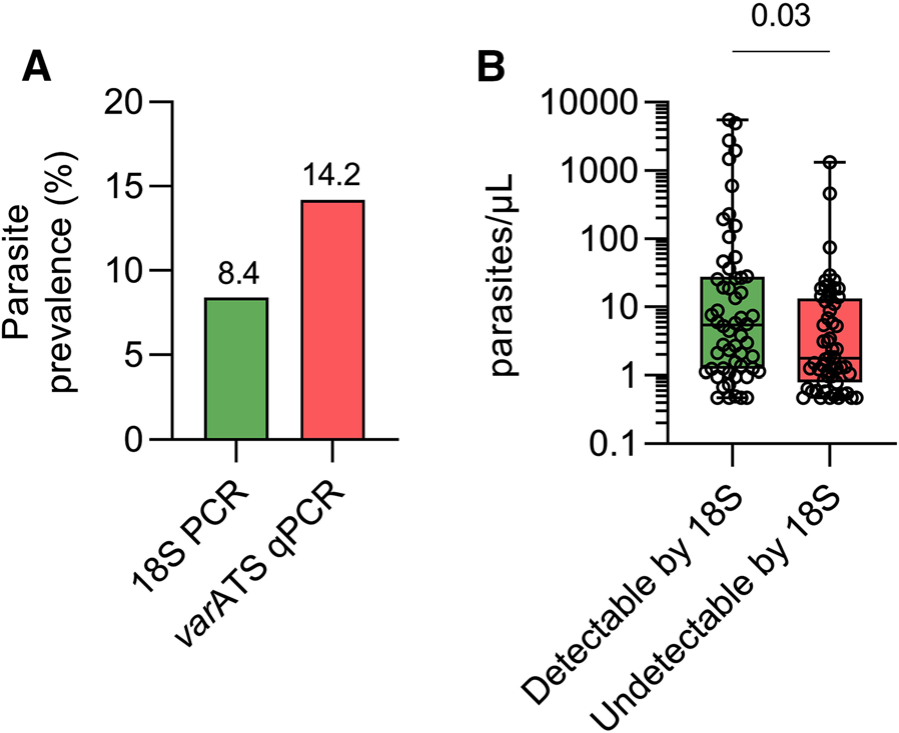
Selecting ***var***ATS qPCR as the gold standard ***P. falciparum*** diagnosis. In subgroup analysis, 788 children were tested for *P. falciparum* by both 18S PCR (positive/negative endpoint PCR) and *var*ATS qPCR. **A** A higher proportion of samples were positive by *var*ATS qPCR (14.2%, n = 112) compared to 18S PCR (8.4%, n = 56). **B** Parasite densities (determined by *var*ATS qPCR) were significantly higher in samples detectable by 18S (median 5.4 parasites/µL, IQR 1.2–27.7, n = 56) than those that were not (median 1.8 parasites/µL, IQR 0.8–13.3, n = 56). Significance determined by Mann-Whitney test

A comparison of molecular diagnostic methods (*var*ATS qPCR and 18S rRNA PCR) was conducted on a subset of samples (*n* = 788) (Fig. 3). For this subset, the proportion of positive subjects was significantly higher with *var*ATS qPCR (*n* = 112, 14.2%) than with 18S PCR (*n* = 66, 8.4%; χ^2^ = 32.06, *p* < 0.0001) (Table 3). Using *var*ATS as the gold standard, 18S rRNA PCR had a sensitivity of 50.0% (95% CI: 40.4–59.6) and specificity of 98.5% (95% CI: 97.3–99.3) in this asymptomatic cohort. The higher prevalence of *var*ATS positive samples in this subgroup analysis (14.2%, Fig. 3A) compared to the larger analysis cohort (10.2%, Fig. 2A) is in part due to timing: only samples collected in the first half of the recruitment period (up to day 29) were tested by 18S rRNA PCR whereas *var*ATS qPCR was used to screen all available samples retrospectively . Interestingly, parasite densities (as determined by *var*ATS qPCR) differed significantly between 18S rRNA detectable samples (median 5.4 parasites/µL, IQR 1. 2-27.7, *n* = 56) and those that were missed by 18S rRNA qPCR (median 1.8 parasites/µL, IQR 0.8–13.3, *n* = 56; *p* = 0.03) (Fig. 3B). This indicates that the 18S rRNA assay was failing to detect a proportion of low-density infections. However, there was considerable overlap in the interquartile range of parasite densities detected by both methods, suggesting that the increased sensitivity of *var*ATS qPCR was not simply due to its lower limit of detection.

**Fig. 4:**
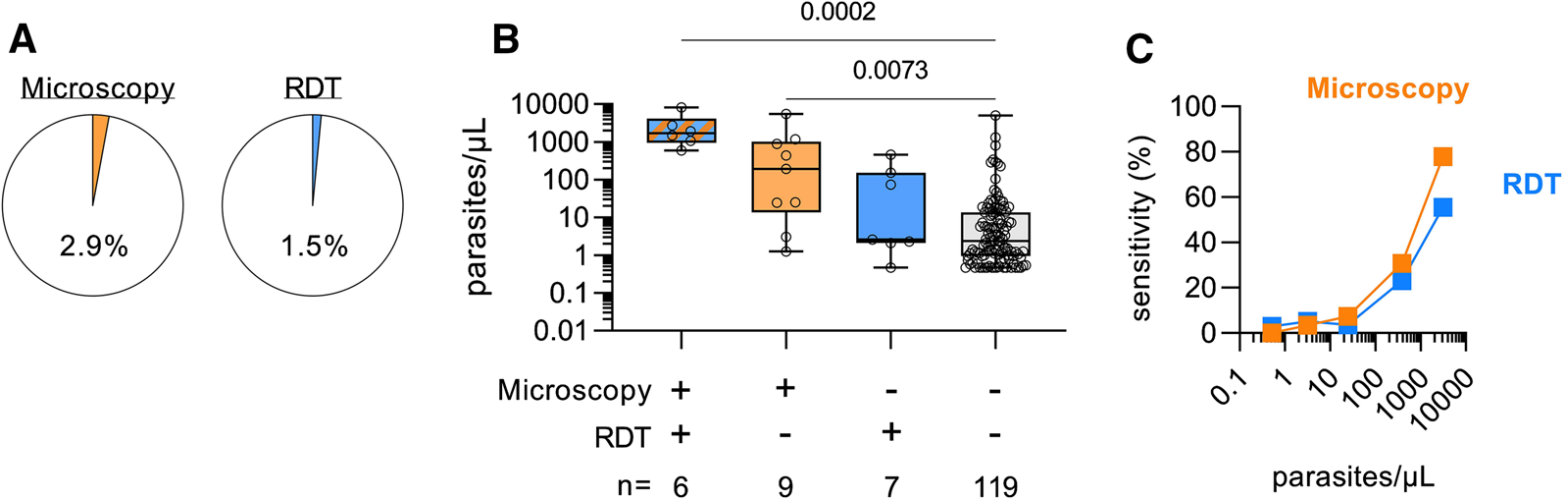
*Plasmodium falciparum* prevalence and density and diagnostic sensitivity of microscopy and RDT. **A** Prevalence of malaria in the cohort as determined by microscopy (left panel, n = 40) and rapid diagnostic test (RDT, right panel, n = 21). **B** Parasite density (as determined by *var*ATS qPCR) in samples testing positive (+) or negative (−) by microscopy and/or RDT. Box and whisker plots with each dot representing an individual, with n value shown. P-values from Dunn’s test with Bonferroni adjustment for multiple pairwise comparisons. **C** Diagnostic sensitivity for microscopy and RDT was calculated amongst *var*ATS positive cases split by parasite density (< 1, 1–10, 11–100, 101–1000 and > 1000 parasites/µL). Data is plotted at the average parasite density observed within each fold range

Consistent with previous reports [21–23], PCR detected large numbers of “subpatent” infections, i.e. infections that were not detected by either microscopy or RDT. Only 1.5% (*n* = 21) of samples were positive by RDT and 2.9% (*n* = 40) by microscopy (Fig. 4A), compared to 10.2% by *var*ATS qPCR. Thus, compared to the gold standard *var*ATS qPCR, the sensitivity of RDT was 9.2% (95% CI: 5.0–15.3) and the specificity was 99.4% (95% CI: 98.7–99.7), and the sensitivity and specificity of microscopy were 10.6% (95% CI: 6.1, 16.9) and 98.0% (95% CI: 97.0–98.7), respectively (Table 3). Unsurprisingly, samples that were positive by all three tests had a significantly higher parasite density (median = 1705 parasites/µL; IQR 1177–2548) than samples that were positive by *var*ATS qPCR alone (median = 2.4 parasites/µL; IQR 1–14) (*p* = 0.0002) (Fig. 4B).

Twenty five samples that were positive by microscopy were negative by *var*ATS qPCR; 21 of these were negative by 18S PCR, *var*ATS qPCR, and RDT suggesting possible mis-speciation by microscopy (as none of the other methods detects species other than *P. falciparum*). One sample was negative by both PCR-based methods, but positive by microscopy and RDT; this suggests an error during the DNA extraction step, as both the PCR and qPCR analyses were conducted using the same extracted DNA. The remaining 3 microscopy-positive/*var*ATS-negative samples were negative by RDT, but were positive by 18S PCR; the precise explanation for this is unclear.

To better understand the diagnostic sensitivity of each assay, the percentage of *var*ATS qPCR positive samples detected by each method was plotted against calculated parasite density (as determined by *var*ATS qPCR) (Fig. 4C). As expected, the sensitivity of both methods increased significantly with parasite density. Neither microscopy nor RDT reliably detected infections with a density below 100 parasites/µL (for both techniques, sensitivity below this parasitaemia threshold was < 7%), and both tests only achieved 50% sensitivity for parasitaemias > 1000 parasites/µL (78% and 56% for microscopy and RDT, respectively). However, given the relatively small number of microscopy and RDT positive samples, precise sensitivity values for these methods should be interpreted with caution.

## Discussion

The Sahel—the climatic and ecological transition zone between the Sahara Desert to the north and the grasslands and tropical forests to the south—was historically a region of intense but highly seasonal malaria transmission. However, malaria infection patterns have been changing in recent decades in response to shifting rainfall patterns and ongoing attempts at malaria control [24, 25], as well as increasing urbanisation [26]. Malaria transmission in The Gambia, where creeping desertification has been evident for many years [27], has been declining for decades and is now regarded as unstable, with marked year-to-year variation in the burden of disease [28]. In 2003, 26% of inpatients in selected hospitals across the country were microscopy positive for malaria, falling to 7% in 2007; malaria-associated deaths in the same hospitals fell by > 90% in the same period [29]. The distribution of infection has also changed, declining much more markedly in western regions (near the coast) than further east (in the Upper River Region, where this study was conducted) [30], and is partly attributed to the changing distribution of a key mosquito vector, *Anopheles gambiae* [31]. As a consequence, clinical immunity is now acquired more slowly during childhood [12, 32] and the burden of both symptomatic and subclinical disease is shifting to somewhat older age groups [33]. It is important, therefore, to periodically reassess patterns of malaria infection in order to most effectively implement malaria control interventions, with particular focus on whether interventions should now include school-age children [34].

In this study, conducted in 2017–2018, approximately 10% of apparently healthy school-aged children in the Upper River Region of The Gambia had a subclinical malaria infection at the start of the dry (low transmission) season. Most infections were detectable only by highly sensitive PCR-based analysis, with both microscopy and rapid diagnostic tests detecting fewer than 10% of qPCR positive infections. Malaria prevalence was also highly heterogeneous across the region, ranging from < 1% to ~ 40% of children in different village clusters. These data are more-or-less in line with a prior study, conducted in April 2014 (i.e. towards the end of the dry season), on the south bank of the Upper River Region. In the 2014 study, community prevalence (among those > 6 months age, regardless of symptoms) was 6.7%, with ~ 60% of infections being subpatent (detectable by 18S rRNA PCR but not by microscopy) [13]. This is likely an underestimate, as 18S rRNA PCR has a sensitivity of < 50% when compared to *var*ATS qPCR. The higher sensitivity of *var*ATS qPCR compared to 18S rRNA PCR (as shown here), and the restriction of the present study to asymptomatic school-aged children, likely explains the higher proportion of subpatent infections reported here (~ 90% vs. ~60%); in line with this, Mwesigwa et al. detected a significantly higher proportion of subpatent infections in children aged 5–15 years than among those < 5 years [13].

Malaria infections are generally classified as being low density or subpatent if they fall below the limit of detection of traditional microscopy and RDT. The median density of asymptomatic infections found here (3.12 parasites/µL, IQR 1.065–19.57) is 6-fold lower than can be detected by the most experienced microscopists (who can detect approx. 5–20 parasites/µL of blood in a thick film [35], equivalent to 0.0001% parasitaemia) and 100-fold lower than the 500 parasites/µL of blood that can be reliably detected microscopically by routine diagnostic laboratories [36].

Rapid diagnostic testing (RDT) can reach a sensitivity of up to 95% for *P. falciparum* when parasite densities are > 100 parasites/µL of blood [37]. In a comparative analysis of > 170,000 individuals, parasite prevalence by microscopy and RDT was highly concordant but with RDT detecting slightly more positive samples than microscopy; microscopy identified approx. 87% of RDT positive cases [38]. In the same analysis [38], RDTs detected 41% of samples that were positive by PCR but concordance between RDT and PCR was low, possibly due to the inclusion in the analysis of studies employing a variety of different PCR assays. Although RDT and microscopy have similar sensitivities, RDTs are quicker, relatively cheap, and are easy to use with minimal training with little variation in results between users. In contrast, microscopy offers advantages in terms of *Plasmodium* speciation and can differentiate asexual stages from gametocytes (which may be detected by antigen-based methods but are no longer clinically relevant to patient disease outcome). Until recently, the standard target for diagnosis of malaria by PCR was 18S ribosomal RNA (rRNA) using a nested PCR approach [39, 40]. There are approximately 5–8 copies of the 18S rRNA sequence per parasite genome [19]. More recently, more abundant molecular targets have been identified that have increased sensitivity of molecular diagnosis: there are between ~ 50 and ~ 150 copies per genome of the *var*ATS gene and 250 copies per genome of the telomere-associated repetitive element 2 (TARE-2), with strain-dependent variation [19]. The *var*ATS qPCR is specific for *P. falciparum* and has been shown to be substantially more sensitive than RDT and microscopy; it can even be used to detect infections from saliva samples [41]. However, strain-dependent variation in gene target copy number can lead to discrepancies in parasite densities measured from genetically variable field isolates; PCR-based amplification of specific sequences of multi-copy genes is currently only able to truly determine parasite density in controlled infections (e.g. during human challenge models), where primers have been designed for known, sequenced, single genotype infections. This is a key diagnostic challenge to be addressed when it comes to molecular analysis of wild-type infections. 

In a cross-sectional study at the peak of the malaria transmission season in The Gambia in 2017, a HRP2-based RDT (HS-RDT) detected 38.4% of samples that were positive by *var*ATS qPCR (with a specificity of 88.5%) [42]. This is higher than the 10% sensitivity reported here, however median parasite densities were likely significantly higher in that study due to the timing of the sample collecting (during ongoing transmission), the age groups recruited and the inclusion of people who may have been symptomatic. Similarly, estimates of 74% sensitivity for RDT and 63% sensitivity for microscopy when compared to *var*ATS in a study in Nigeria likely reflect the preponderance of symptomatic cases and the high median parasitaemia (6,689 parasites/µL as determined by *var*ATS qPCR) [42–44]. In support of this contention, in a study in a high transmission setting in western Kenya, RDT sensitivity against *var*ATS qPCR was 36% overall but only 25.5% in asymptomatic individuals and 15.8% in those aged more than 15 years [44] suggesting that median parasite density is an important variable in reliability of RDT tests, and that the lower limit of detection in these tests is more representative of symptomatic malaria rather than subclinical parasitaemia.

Importantly, subclinical/subpatent malaria infections are not necessarily benign; they have been associated with an increased likelihood of subsequent symptomatic disease, anaemia, bacterial coinfections and impaired cognitive development, as well as maintaining a reservoir of ongoing transmission [15]. Persistent, low density infections have been linked to low grade inflammation, specifically raised plasma concentrations of C-reactive protein [45], IFN-γ [46, 47], CXCL1 [48], IL-10 [46, 49], and IL-6 [47, 49]. Low grade anaemia may also be more common in those with subpatent infections than among those without detectable infection [46] although this is not a universal finding [16, 50]. Differences between studies in the immunological and haematological status of people with asymptomatic/subpatent infections likely reflect different epidemiological and cohort characteristics and/or methods by which the uninfected control group is defined. For example, in a longitudinal analysis, raised concentrations of pro-inflammatory markers in children who had resolved a subpatent malaria infection within the previous 6 weeks [16]; these children would have been deemed uninfected in a cross-sectional study.

In summary, ~ 10% of school-aged, otherwise healthy, Gambian children carried extremely low-density malaria infections into the dry (low transmission) season. These infections were detectable using an ultra-sensitive PCR diagnostic method (*var*ATS qPCR) but were not reliably detected by a more conventional PCR assay (18S rRNA PCR), and the overwhelming majority of these infections were missed by both RDT and microscopy. Understanding the duration of these infections, their contribution to sustained transmission (maintaining a reservoir of infection and transmissible gametocytes between rainy seasons) and their physiological consequences (in terms of childhood development and acquired immunity) will require longitudinal studies with frequent resampling throughout the year, bridging high, low and non-transmission seasons. Sustained malaria control, with the ultimate goal of malaria elimination, may be difficult to achieve without detailed understanding of low-density *P. falciparum* infections.

**Table 1:** Cohort characteristics

	Survey Cohort (*n* = 1650)	Analysis cohort (*n* = 1381)	Survey vs. analysis (*p* value*)	*var*ATS-negative (*n* = 1240)	*var*ATS-positive (*n* = 141)	PCR + vs. – (*p* value*)
Sex (males)	n = 808 (49%)	n = 704 (51%)	0.13	n = 620 (50%)	n = 84 (60%)	0.03
Age (y)	10 (9–12)	10 (9–12)	0.75	10 (9–12)	11 (9–12)	0.16
Weight (kg)	28.2 (24.4–34)	28.0 (24.3–33.6)	0.55	28.0 (24.4–33.5)	27.7 (24-34.7)	0.80
Height (m)	1.37 (1.31–1.46)	1.37 (1.31–1.46)	0.74	1.37 (1.31–1.45)	1.36 (1.29–1.47)	0.70
Temperature (°C)	36.9 (36.7–37.1)	36.9 (36.7–37.1)	0.99	36.9 (36.7–37.1)	36.9 (36.7–37.2)	0.39
Pulse (bpm)	98 (91–107)	98 (90–107)	0.65	98 (90–107)	98 (90–104)	0.17
Oxygen saturation (%)	98 (96–98)	98 (96–98)	0.95	98 (96–98)	98 (96–99)	0.57
Day post study start	28 (15–33)	28 (15–33)	0.06	28 (12–33)	16 (15–28)	0.0005

Demographic, anthropomorphic and clinical data for the entire survey cohort and the analysis cohort, sampled from 11 Dec 2017 to 18 Jan 2018 (38 days) and by malaria status, as determined by *var*ATS qPCR. Median values shown with interquartile range (Q1–Q3)

*P-values obtained from a chi-square test (binary data) or Mann–Whitney test (continuous data)

**Table 2:** Distribution of *P. falciparum* prevalence by village cluster

Village cluster	Cluster sample no. (n)	Village number	Village name	Day of visit^a^ (range)	Total participants (n)	*var*ATS-negative (n)	*var*ATS-positive (n)	*P. falciparum* prevalence (%)
i	93	1	Koro Jula Kunda	8–9	36	35	1	4
		2	Koro Numu Kunda	8	26	23	3	
		3	Sare Talata	7	8	8	0	
		4	Busura Alieu	7	23	23	0	
ii	206	5	Sare Kokeh	10	4	4	0	3
		6	Sare Dembel Jawo	11	5	5	0	
		7	Kosemari	3–9	30	30	0	
		8	Bakadagie	10–11	115	113	2	
		9	Hella Kunda	12	39	37	2	
		10	Sare Mamudu	12	13	10	3	
iii	81	11	Sotuma Sere	15	81	49	32	40
iv	85	12	Numuyel	16	55	25	30	36
		13	Gambisara	0–7	30	29	1	
v	64	14	Tamba Sansang	23	64	50	14	22
vi	80	15	Kundam MaFatty	29	80	73	7	9
vii	123	16	Dandu	28	12	10	2	13
		17	Sanunding	24–28	107	93	14	
		18	Kunkandy	26	3	3	0	
		19	Kulinto	26	1	1	0	
viii	78	20	Fass Bajong	24	38	38	0	1
		21	Baniko Kekoro	25	40	39	1	
ix	106	22	Dingiri	30–31	106	105	1	1
x	163	23	Sinchang Jabo	12	10	7	3	8
		24	Suduwol	33–35	117	110	7	
		25	Kumbul	33–35	36	33	3	
xi	243	26	Garawoll	36–37	173	171	2	2
		27	Sami Kuta	32	25	24	1	
		28	Sami Koto	32	45	43	2	
xii	59	29	N’yamanari	38	59	51	8	14

Study enrolment was performed by passive recruitment at village community centres. Villages were clustered, post-hoc, into twelve clusters by spatial proximity (additional detailed map data found in Additional file 1: Fig. S1). The median number of individuals per cluster was 89 (IQR 80–133) and the median number of individuals per village was 36 (IQR 13–64)

^a^Day from village visit from the study commencement, 11 Dec 2017, with prevalence over time shown in Fig. 2B

**Table 3:** Comparison of *P. falciparum* diagnostic results

		RDT (*n* = 1381)		Microscopy (*n* = 1381)		18S PCR (*n* = 788)	
		Positive	Negative	Positive	Negative	Positive	Negative
* var ATS * *qPCR*	Positive	13	128	15	126	56	56
	Negative	8	1232	25	1215	10	666
Specificity (95% CI)		99.4% (98.7–99.7)		98.0% (97.0–98.7)		98.5% (97.3–99.3)	
Sensitivity (95% CI)		9.2% (5.0–15.3)		10.6% (6.1–16.9)		50.0% (40.4–59.6)	
Risk ratio^a^ (95% CI), *p* value		1		1.2 (0.66–2.0) 0.80		5.6 (3.1–10.1) < 0.0001	

Using *var*ATS qPCR as the ‘gold standard’ comparator, assay results are shown for RDT and microscopy in the analysis cohort (*n* = 1381) and for 18S PCR in the subgroup analysis (*n* = 788). Assay sensitivity was calculated as the number of assay positives detected divided by the number of true total positives (i.e. *var*ATS positive). Assay specificity was calculated as the number of assay negatives detected divided by the number of true total negatives (i.e. *var*ATS negative)

^a^Risk Ratios (with confidence intervals) and *p*-values comparing sensitivity estimates obtained from McNemar’s test (comparator = RDT)

## Supplementary information

**Additional file 1: Fig. S1**. Distribution of P. falciparum prevalence by village within clustered data. Related to Fig. 2. For villages which were clustered, parasite prevalence is shown for individual villages within each. Clusters were assigned to them by relative distance, as seen in each inset. The diameter of the pie chart is relative to the number of patients sampled, with detailed sampling numbers given in Table 2.

## Data Availability

The raw data supporting the conclusions of this article will be made available by the authors, without undue reservation, to any bona fide researcher.

## Abbreviations

PCR: Polymerase chain reaction
18S rRNA: 18S (Svedberg units) ribosomal ribonucleic acid
*var*ATS: var gene acidic terminal sequence
IQR: Interquartile range
RDT: Rapid diagnostic test
URR: Upper River Region (of The Gambia)
LLOQ: Lower limit of quantification
