# Supplementary material for: Dry season prevalence of Plasmodium falciparum in asymptomatic gambian children, with a comparative evaluation of diagnostic methods
Source: Malar J. 2022 Jun 7;21:171. doi: 10.1186/s12936-022-04184-9 (PMC9172138; doi:10.1186/s12936-022-04184-9)
Supplement: Supplementary file 1 — Additional file 1: Fig. S1. Distribution of P. falciparum prevalence by village within clustered data. Related to Fig. 2. For villages which were clustered, parasite prevalence is shown for individual villages within each. Clusters were assigned to them by relative distance, as seen in each inset. The diameter of the pie chart is relative to the number of patients sampled, with detailed sampling numbers given in Table 2. [file 12936_2022_4184_MOESM1_ESM.docx]

**Additional file 1**

**Dry season prevalence of *Plasmodium falciparum* in asymptomatic Gambian children, with a comparative evaluation of diagnostic methods.**

Jason P. Mooney^1#^; Sophia M. DonVito^1^; Maimuna Jahateh^2^; Haddy Bittaye^2^; Christian Bottomley^3^; Umberto D'Alessandro^2^; Eleanor M. Riley^1^

^1^ Institute of Immunology and Infection Research, School of Biological Sciences, University of Edinburgh, Edinburgh, United Kingdom.

^2^ Medical Research Council Unit in The Gambia at the London School of Hygiene and Tropical Medicine, Fajara, The Gambia.

^3^ Department of Infectious Disease Epidemiology, London School of Hygiene and Tropical Medicine, London, United Kingdom.

^#^Correspondence address: Jason P Mooney PhD, Institute of Immunology and Infection Research, School of Biological Sciences, Ashworth Laboratories, Kings Buildings, Charlotte Auerbach Rd, Edinburgh, EH9 3FL, UK

E-mail: [jason.mooney@ed.ac.uk](mailto:jason.mooney@ed.ac.uk)


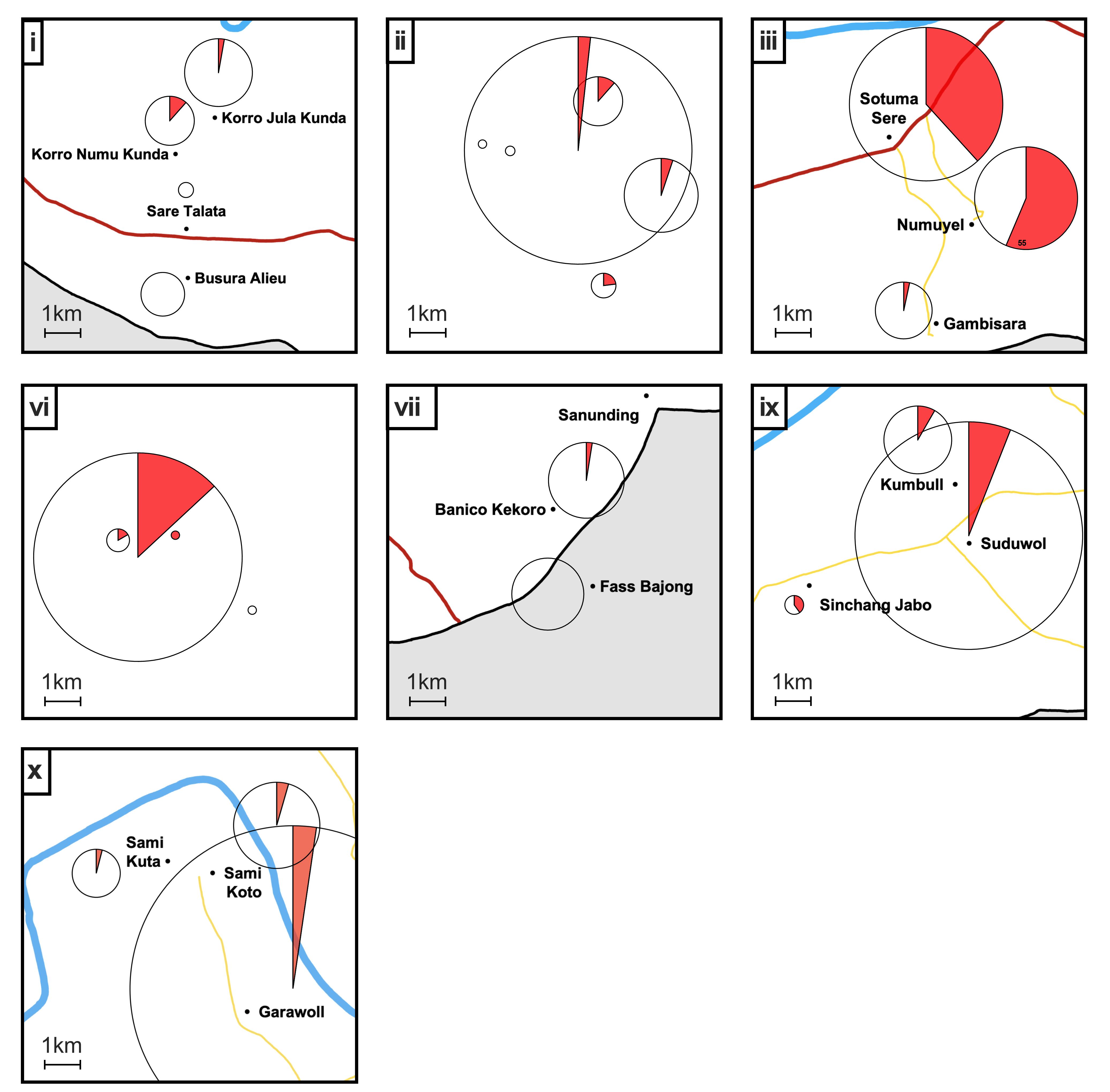


**Supplemental Figure 1:** **Distribution of *P. falciparum* prevalence by village within clustered data.** Related to Figure 2. For villages which were clustered, parasite prevalence is shown for individual villages within each. Clusters were assigned to them by relative distance, as seen in each inset. The diameter of the pie chart is relative to the number of patients sampled, with detailed sampling numbers given in Table 2.
